# Hens That Exhibit Poorer Feed Efficiency Produce Eggs with Lower Albumen Quality and Are Prone to Being Overweight

**DOI:** 10.3390/ani11102986

**Published:** 2021-10-16

**Authors:** Doreen Onyinye Anene, Yeasmin Akter, Peter Campbell Thomson, Peter Groves, Sonia Liu, Cormac John O’Shea

**Affiliations:** 1 Department of Animal Science, University of Nottingham, Loughborough LE12 5RD, UK; doreen.anene@nottingham.ac.uk; 2 Poultry Research Foundation, University of Sydney, Camden, NSW 2570, Australia; yeasmin.akter@sydney.edu.au (Y.A.); peter.groves@sydney.edu.au (P.G.); sonia.liu@sydney.edu.au (S.L.); 3 School of Life and Environmental Sciences (SOLES), University of Sydney, Camden, NSW 2570, Australia; peter.thomson@sydney.edu.au; 4 Department of Bioveterinary and Microbial Sciences, Technological University of the Shannon, Midlands Midwest, N37 HD68 Athlone, Ireland

**Keywords:** egg quality, laying hens, performance traits, feed efficiency, amino acids

## Abstract

**Simple Summary:**

The contemporary hybrid layer is an efficient producer of eggs, which is a source of high-quality nutrients. However, there is a lack of scientific knowledge on how feed efficiency (FE), an important measure of hen productivity, differs between individual hens during laying life and its association with other hen performance and egg quality traits. This study sought to investigate the production traits, egg composition and quality of laying hens in mid-lay when ranked based on FE in early-lay. The results showed that feed to egg conversion ratios (FCR) exhibited in early-lay were maintained until at least 40 weeks, with feed intake being the major driver of the difference in FE, not the mass of the egg. Further, hen and egg quality traits are associated during mid-lay, with high feed efficient hens having a lower body weight but producing eggs whose albumen has a higher height, Haugh unit and amino acid concentration. These results may provide important information to poultry breeders and egg producers towards improving the economics of egg production and generally improve management decision making, which is usually made based on accepting the expected average performance of a cohort of animals.

**Abstract:**

Feed efficiency (FE) is an important measure of productivity in the layer industry; however, little is known about how FE differs between individual hens during the egg-laying cycle and the implications for egg quality parameters. Individual 25-week-old ISA Brown hens were observed for 42 days, ranked into three FE groups (*n* = 48 per High (HFE), Medium (MFE) and Low (LFE) FE groups and then monitored later in the laying cycle from 35–40 weeks. The groups exhibited different feed to egg conversion ratios (*p* < 0.001) from 35–40 weeks. Average daily feed intake and body weight were highest (*p* < 0.001) in the LFE group compared to the MFE and HFE groups, while albumen height, Haugh unit and amino acid concentrations of the albumen were significantly higher in the HFE groups compared to the LFE cohort (*p* < 0.001). This study concludes that FE status established in early lay is a stable variable until at least 40 weeks of age, and overweight, mid-laying hens that had poor FE produced inferior egg albumen quality measurements and composition. The distinct traits of the highly efficient hens and the poor feed efficient hens may provide important information to improving productivity in egg production.

## 1. Introduction

The feed to egg conversion ratio (FCR) is an important marker of feed efficiency (FE) in the egg industry, as it captures the efficiency with which the quantity of feed consumed by laying hens is converted to egg mass. First described in 1941, the FCR is influenced by genetics [1,2,3], physiological, environmental and management factors [4,5], and has improved by 42% from 3.44 in the 1960s to an average of 1.98 in 2010 [6]. An improved (lower) FCR means less feed is required to produce eggs, thus making it a trait that influences breed selection, overall profitability [7] and a reduced carbon footprint on the environment [8,9,10]. Despite its improvements and importance, the inherent variation in FCR between individual hens has received less attention due to the challenges in profiling feed intake, egg production and FCR between individual hens within a flock. Studies have shown that variation in the FE between individual hens is substantial [11,12,13]; however, the influence of this variation on other hen performance characteristics and egg quality traits is not well understood.

Previous work investigating different body weight groups on performance and egg quality showed that light-weight hens between 1400 g to 1500 g on a flock basis consumed less feed, produced lighter eggs, had a lower (better) FCR and a higher Haugh unit score compared to heavier weight birds [11]. The limitation with assessing production variables on a flock basis is that important variations among individuals are not captured and the subsequent inconsistency in voluntary feed intake and hen body weight can result in a poor uniformity in egg production as well as external and internal egg quality characteristics [14]. In addition, the absence of information about individual hens negatively influences nutrition and management decisions, which are usually made, by necessity, based on the expected average performance of a cohort of animals. A few studies on the FE of individual hens [2,15] reported that laying hens which were classified as efficient feed converters, consumed less feed per gram of egg mass and were lighter than intermediate and inefficient hens. These studies were, however, focused on white-shelled hens, and there is no information about how early in the laying phase differences in FCR exist between individual hens and whether those differences may remain at later periods in lay.

The quality of the egg is evaluated by several parameters in the albumen, yolk, and shell; however, the association of FCR with measurements of egg quality is relatively unknown. A recent preliminary study showed that in a flock of individually housed hens on the same dietary and environmental conditions, differences in the body weight and feed efficiency of ISA Brown hens aged 55 weeks old were associated with egg quality variables [12]. That study reported that hens profiled for having poor (high) FCR had a lower Haugh unit, a heavier yolk and a greater concentration of yolk saturated fatty acids when compared with those described as excellent (low) FCR. The limitation of that study was that the investigation was carried out on hens approaching the later stages of the laying cycle; thus, there is no information on if or how early in lay these differences between individual hens are established. 

Despite genetic improvements which have produced hybrid hens with high egg productivity, there is a scarcity of scientific knowledge on the onset and persistency of the differences that exist in individual hen FE and its resulting associations with other hen performance traits and egg quality characteristics. Therefore, the objectives of this study were to evaluate the hen performance traits, nutrient digestibility and egg quality characteristics of individual ISA Brown hens in the mid-laying stage of egg production (35–40 weeks), ranked based on retrospective feed efficiency performance during the early-lay period (25–30 weeks). 

## 2. Materials and Methods

### 2.1. Ethics Approval

The experiment was conducted at the Poultry Research Foundation (PRF) layer rearing facility within the University of Sydney Camden Campus, New South Wales, Australia. The procedures and activities conducted in this study were approved by the University of the Sydney Animal Ethics Committee, protocol number AECL 2017/1212 and in accordance with the 8th edition of the Australian Code for the care and use of animals for experimental purposes [16].

### 2.2. Experimental Diet

A wheat soybean meal-based mash diet containing 16.30% crude protein and 2750 kcal/kg of gross energy was formulated according to the ISA Brown management guide [17] to meet the nutritional requirements of the birds [13]. The same diet was supplied to all experimental birds in individual feeding troughs placed in front of the cages. Fresh diets were measured out weekly and offered, and at the end of the week, feed not consumed was taken out, measured, and recorded.

### 2.3. Onset of Lay, Egg Weight at First Lay

A total of 455 pullets aged 16 weeks were obtained from a certified grower farm, weighed individually and randomly assigned to 25 × 50 × 50 cm single multitier metal layer cages in the layer research facility. The shed temperature was controlled around 21–24 °C, and photoperiod cycles of 16 h light and 8 h darkness were maintained, as recommended by the ISA Brown management guide [17]. All hens were watered by automatic nipples installed in the cages and were fed the experimental diet on an ad libitum basis. From 16 weeks up to 24 weeks, all hens were monitored for the date of first oviposition: the day when the first egg for each individual hen was laid, and the egg laid was weighed and recorded. 

### 2.4. Preliminary Study, 25–30 Weeks of Age

When the hens were 24 weeks of age, they were weighed individually, and the weight was recorded as initial body weight (IBW). Data collection started from 25 weeks up to 30 weeks (estimated as the early-lay stage), lasting a total of 42 days. During this period, egg output and egg weight were recorded daily, and feed intake was measured weekly. The weekly feed (to egg) conversion ratio (FCR) was calculated as the grams of feed consumed per gram of egg mass. All hens were weighed individually at 30 weeks and recorded as final body weight (FBW). Based on the FCR from the preliminary study period, the experimental hens were ranked into quintiles, and the hens in the first, third and fifth quintile were classed into three groups, namely: High feed efficiency (HFE), medium feed efficiency (MFE) and low feed efficiency (LFE). HFE hens had an average FCR of 1.83; MFE, 2.05; LFE, 2.39. The performance and egg quality measurements of the wider cohort of hens from this early lay phase has recently been published [13].

### 2.5. Hen Performance, 35–40 Weeks of Age

At 35 weeks of age (estimated as the mid-laying stage), a total of 144 hens (*n* = 48 hens per FE group) from the preliminary study were randomly assigned to the three FE groups. Each hen was housed individually and distributed randomly throughout the upper, middle, and bottom levels of three-tier layer metal cages. The experiment lasted for 42 days, between 35–40 weeks of age. All hens were monitored individually, and the following hen performance parameters were collected: hen IBW was measured at 30 weeks old and FBW at 40 weeks of age using an electronic scale with a digital output. Body weight change was recorded as the difference between initial and final body weight. Eggs produced per hen were recorded daily, and the egg production (EP) was calculated in percentage as (total number of eggs produced in seven days × 100)/7. Feed was distributed once per week and topped-up as required, and refused feed was removed and weighed once weekly. The average daily feed intake (ADFI, g) was calculated as (weekly feed offered − weekly feed unconsumed)/7. Egg weights (g) were recorded daily per hen using a digital weighing balance, and the average egg mass per hen (g) was calculated as: EP × EW/100; where EP = Egg production, and EW = average egg weight.

### 2.6. External Egg Abnormality Score

All eggs collected were assessed individually for external abnormalities during the egg collection for the preliminary period (25–30 weeks) and mid-lay stage (35–40 weeks). The egg score was the number of eggs with abnormalities that were either double-yoked, dirty, blood-stained, shell-less, thin, or soft-shelled, cracked, odd-shaped, broken, or frosty. The number of abnormalities in individual hen eggs was totaled, analysed and compared on an FE basis across the two experimental periods.

### 2.7. Egg Quality Analysis

A total of 45 hens from the study (*n* = 15 per FE group) were randomly selected and marked. Once every week for the duration of the study, their eggs were collected and subjected to internal and external egg quality assessments.

#### 2.7.1. External Quality

After collection, eggs were weighed individually using an electronic balance with a digital readout to the precision of 0.01 g. Egg height (mm) and width (mm) were measured using an electronic Vernier caliper (Kincrome, Australia). The egg shape index was calculated as egg width divided by egg height, multiplied by 100. The eggs were carefully broken out on a flat, levelled glass surface on a metal stand with a reflective mirror. The eggshells were carefully washed, air-dried for 72 h and weighed with a digital scale to a precision of 0.0001 g. Eggshell membranes were removed, and eggshell thickness (mm) was measured at three regions (pointy end, equator and blunt end) using a digital caliper to the nearest 0.001 mm; the mean obtained from the three values was recorded as the average shell thickness. The eggshell percentage was calculated as the eggshell weight multiplied by 100 and divided by the egg weight. 

#### 2.7.2. Internal Quality

The thick albumen and yolk widths were measured with a digital Vernier caliper at the equator while still combined. Albumen height was measured using an albumen height gauge with an electronic display (Technical Services and Supplies, York, UK). The probe on the gauge detects and measures the height of the thick albumen when an egg is broken out onto a flat surface. Using a plastic scraper, the thick and thin albumen was separated from the yolk, collected into a clean container and weighed. The yolk height was measured using a tripod micrometer (B.C. Ames Co., Waltham, MA, USA), and yolk colour was determined using a DSM colour Yolk fan (DSM, Switzerland 2005), on a scale from 1 through 16 units. The yolk was collected in a clean container and weighed using an electronic balance with a digital readout. The albumen and yolk pH were then measured using a calibrated pH meter. The Haugh unit, was derived using the formula 100 × log10 (h − 1.7 × w0.37 + 7.6), where h = albumen height (mm), and w = egg weight (g) [18]. The albumen and yolk ratios were calculated from the individual weights of the albumen and yolk. The yolk index was calculated as yolk height/yolk width × 100, and the albumen index was calculated as albumen height/albumen width × 100. Fresh eggs were collected from the same hens the following day, and eggshell breaking strength (g) was measured using a texture analyser (Perten TVT 6700, Stockholm, Sweden), fitted with a cylindrical probe 75 mm in diameter, as the peak force which must be applied to the egg before it fails.

### 2.8. Quantitative Amino Acid Concentration Analysis

A total of 20 eggs were randomly collected (*n* = 10 per HFE and LFE group), their albumen separated and based on their dry matter, analysed for concentration of 15 amino acids. The analysis of amino acid contents of the egg albumen was done at the Australian Proteome Analysis Facility (APAF), within Macquarie University, New South Wales, Australia. After first performing acid hydrolysis, the concentrations of amino acids were determined using pre-column derivatisation amino acid analysis with 6-aminoquinolyl-N-hydroxysuccinimidyl carbamate (AQC), followed by separation of the derivatives and quantification by reversed-phase high-performance liquid chromatography (HPLC) [19,20]. Amino acids were detected by UV absorbance (260 nm). Approximately 150 mg of sample was hydrolysed in 20% HCl for 24 h at 110 °C. Following hydrolysis, an internal standard (DL-Norvaline and α aminobutyric acid; AABA) was added to each sample. Following a 1:25 dilution in milliQ water, 10 μL of the solution was derivatised using an AccQ Tag Ultra Derivatization Kit (Waters Australia). HPLC analysis was based on the method of Cohen (2001) [20], but adapted for use with an ACQUITY Ultra Performance LC (UPLC, Waters Corp., Milford, MA, USA) system. The column employed was an ACQUITY UPLC C18 1.7 μm, 100 mm column (Waters) with detection at 260 nm and a flow rate of 0.7 mL/min. This enabled a 10.2 min. analysis time per sample. Samples were analysed in duplicate, and the results averaged. The use of HCl as the hydrolysis reagent converts the amino acids asparagine and glutamine to their acid forms, aspartic acid and glutamic acid, respectively. In the presence of HCl, the amino acid tryptophan is destroyed while cysteine is partially destroyed. Therefore, quantitation for cysteine and tryptophan could not be undertaken by this hydrolysis method.

### 2.9. Nutrient Digestibility

At 45 weeks of age, 60 hens, *n* = 20 hens per FE group, were randomly selected to be used for a 72-h nutrient digestibility study. The diet was measured out and fed to the selected birds on an ad libitum basis for a total of 8 days, comprising a 5-day adaptation period to the diet and then a 3-day excreta collection period. Aluminium trays were laid underneath the cages of selected birds to capture total excreta (g) over a period of 72 h for the determination of apparent metabolisable energy (AME, MJ/kg) and nitrogen retention (N%). At the end of the collection period, total feed intake was calculated, and the total excreta collected was then joined per feed efficiency group, thoroughly homogenised, weighed and stored in a freezer at −20 °C. From the homogeneous mass, known quantities of excreta samples were dried in crucibles in a forced ventilation oven at 70 °C for 72 h to constant weight to determine DM. Excreta were freeze-dried for gross energy and N analysis. Wet excreta dry matter (DM) was calculated by correction for the loss of moisture during both freeze- and oven-drying. Feed samples were also dried and analysed for crude protein (CP), energy efficiency (EE) [21], and results were expressed on a DM basis. From the results of laboratory analysis information of DM intake, metabolisable coefficients of DM, CP, gross energy (GE), value of apparent metabolisable energy (AME) and AME corrected for nitrogen retention (AMEn) of the feed and nitrogen retention per day were obtained. 

DM digestibility (%) was calculated as:

$$\frac{{\mathrm{Dry}\;\mathrm{matter}\;\mathrm{intake}}_{\mathrm{feed}}-{\mathrm{Dry}\;\mathrm{matter}\;\mathrm{output}}_{\mathrm{excreta}}}{{\mathrm{Dry}\;\mathrm{matter}\;\mathrm{intake}}_{\mathrm{feed}}}\times 100$$

Gross energy was analysed using an adiabatic bomb calorimeter (IKA Werke, C7000, GMBH and CO., Staufen, Germany), and the GE retention was calculated as:

$$\mathrm{GE}\;\mathrm{Retention}\;\left(\mathrm{MJ}/\mathrm{kgDM}\right)=\frac{\mathrm{Dry}\;\mathrm{matter}\;\mathrm{intake}\times {\mathrm{GE}}_{\mathrm{diet}}-\mathrm{Dry}\;\mathrm{matter}\;\mathrm{output}\times {\mathrm{GE}}_{\mathrm{excreta}}}{\mathrm{Dry}\;\mathrm{matter}\;\mathrm{intake}\times {\mathrm{GE}}_{\mathrm{diet}}}$$

Nitrogen (N) content of diets and excreta were determined using an elemental analyser (Leco Corporation, St Joseph, MI, USA), and N retention was calculated from the following equation:

$$N\;\mathrm{Retention}\;\left(\%\right)=\frac{\mathrm{Feed}\;\mathrm{intake}\times N_{\mathrm{diet}}-\mathrm{Excreta}\;\mathrm{output}\times N_{\mathrm{excreta}}}{\mathrm{Feed}\;\mathrm{intake}\times N_{\mathrm{diet}}})$$

The AME values of the diets on a DM basis were calculated from the following equation: The AME was determined using the total collection method of Bourdillon et al. [22]. The AME values were converted to AMEn (AME for zero N retention in body). The AME and AMEn were calculated as:

$$\mathrm{AME}=\frac{{\mathrm{DM}\;\mathrm{intake}}_{\mathrm{diet}}\times {\mathrm{Gross}\;\mathrm{energy}}_{\mathrm{diet}}-\mathrm{DM}\;\mathrm{output}\times {\mathrm{Corrected}\;\mathrm{GE}}_{\mathrm{excreta}}}{{\mathrm{DM}\;\mathrm{intake}}_{\mathrm{diet}}}$$

AMEn = AME − 0.036 × N Retention

### 2.10. Statistical Analysis

Hens were ranked based on their FCR performance from 25–30 weeks, into quintiles, and three FE groups were formed from the lowest FCR (1st quintile), average FCR (3rd quintile) and highest FCR (5th quintile). Data were tested by one-way ANOVA using the PROC GLM procedure of the SAS University Edition software (SAS Institute Inc., Cary, NC, USA) with FE group as the factor. The individual hen within each FE group served as the experimental unit. Differences among least squares means were computed using the pdiff statement in SAS. The effect of FE group on the external egg abnormality score count data were analysed using a Poisson generalised linear model using the GLM function in R [23]. The emmeans package in R was used to generate the equivalent of least-square means ± standard errors. All results are presented as least-square means ± standard error of the mean (SEM), except the data from the preliminary ranking period. The *p*-value that denotes statistical significance was set as <0.05.

## 3. Results

### 3.1. Hen Performance

The performance of early-laying hens (*n* = 48 per FE group) between 25–30 weeks and mid-laying hens (*n* = 48 per FE group) between 35–40 weeks of age from varying FE groups are presented in Table 1. The hen groups maintained their FE rankings from the early-laying stage up to 40 weeks of age. In the mid-laying stage, average daily feed intake (ADFI) was significantly different across the three groups, (*p* < 0.001). The hens in the high feed efficiency (HFE) cohort consumed the least amount of feed, 113 g, followed by the medium feed efficiency (MFE) group, 118 g and the low feed efficiency (LFE) group consumed the most amount of feed daily, 128 g. Final body weight was significantly different across the three FE groups (*p* < 0.001), with hens in the LFE groups weighing the most, followed by the MFE group and HFE hens weighing the least. The egg mass was higher (*p* = 0.01) in the HFE group compared to the LFE group. 

### 3.2. Onset of Lay and Egg Weight at First Lay

**Figure 1:**
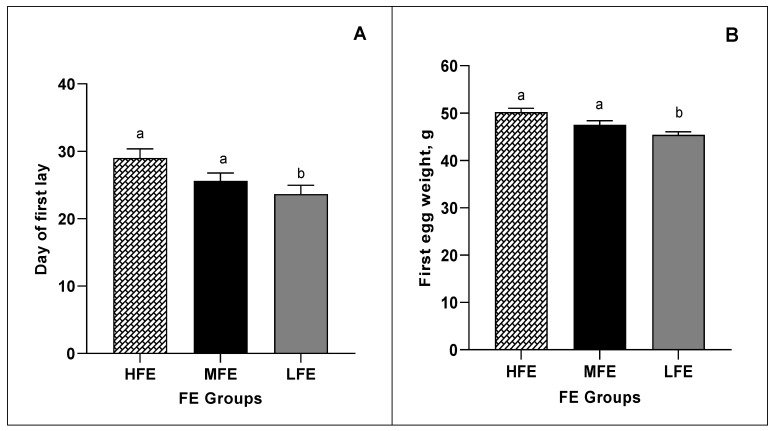
(**A**) Day of the first egg laid by individual ISA Brown hens ranked based on FE (*n* = 48 per FE group) across a 57- day onset of the lay window. (**B**) The weight of the first egg of individual ISA Brown hens ranked based on FE (*n* = 48 per FE group). FE: Feed efficiency; HFE: High feed efficiency; MFE: Medium feed efficiency; LFE: Low feed efficiency. ^ab^ Columns not sharing a common superscript are significantly different at the 5% level of significance.

Figure 1A presents the number of days until the first oviposition, while Figure 1B shows the weight of the first egg laid by individual ISA Brown pullets from between 16 weeks until 24 weeks old, across the three FE groups (*n* = 48 per FE group). All pullets laid their first egg within a 57-day window, which is the difference in days between the first oviposition and the last first oviposition of the entire group. The average onset of lay (day first egg was laid) for hens in the HFE cohort was day 29, which was later (*p* = 0.01) than the LFE hens that on average laid six days earlier, on about day 23. However, the weight of the first egg laid was significantly heavier in hens from the HFE cohort 50.2 g vs. 45.4 g ± 0.82; *p* = 0.0009) compared to hens from the LFE cohort. 

### 3.3. Egg Abnormality Score

**Figure 2:**
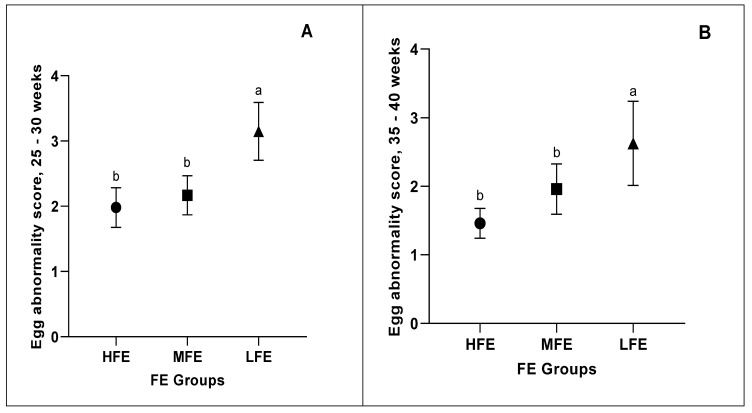
(**A**) Average egg abnormality score of individual ISA Brown hens aged 25–30 weeks based on differences in their feed efficiencies, *n* = 48 hens per FE group. (**B**) Average egg abnormality score of individually housed ISA Brown hens aged 35–40 weeks based on differences in their feed efficiencies, *n* = 48 hens per FE group. FE: Feed efficiency; HFE: High feed efficiency; MFE: Medium feed efficiency; LFE: Low feed efficiency. ^ab^: Means not sharing a common superscript are significantly different at the 5% level of significance.

**Figure 3:**
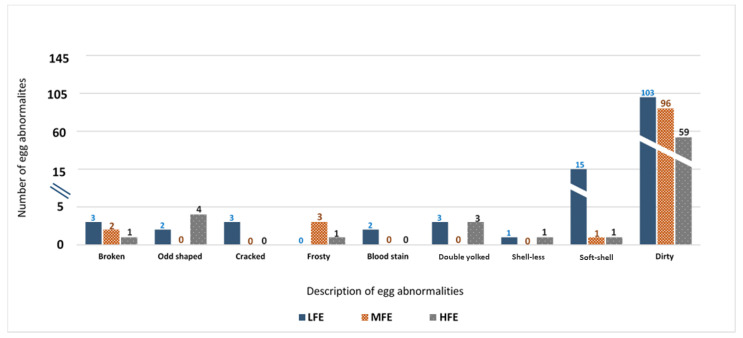
The breakdown of abnormalities in eggs from individual ISA Brown hens of varying feed efficiencies in the mid-laying stage aged between 35–40 weeks (*n* = 48 per high, medium, and low FE groups). HFE: High feed efficiency; MFE: Medium feed efficiency; LFE: Low feed efficiency. A total of 70 abnormal eggs were collected from the HFE group for the entire experimental period, while a total of 94 and 126 were collected from the MFE and LFE groups, respectively, for the 42-day experimental period.

The average egg abnormality score for individual ISA Brown hens in the early-laying period (25–30 weeks) and the mid-laying period (35–40 weeks) are presented in Figure 2A,B, respectively. The average egg abnormality score was higher (*p* < 0.01) in the LFE cohort compared to the HFE group in both experimental periods. The description and composition of egg abnormalities collected from hens in the mid-laying stage (35–40 weeks) for the HFE, MFE and LFE groups are described in Figure 3. Briefly, dirty eggs constituted the highest percentage of egg abnormalities observed and was highest in the LFE cohort, with 103 dirty eggs followed by the MFE group with 96 dirty eggs and was least in the HFE group, with 59 dirty eggs over the 42-day experimental period. The number of soft-shelled eggs was also highest in the LFE group, as a total of 15 soft-shelled eggs were collected from the LFE group across the 42-day experimental period of mid-laying hens, compared to one each collected from the MFE and HFE groups. 

### 3.4. Egg Quality

The albumen, yolk and shell quality characteristics of eggs from hens of different FE groups between 35–40 weeks are presented in Table 2. Briefly, albumen height, albumen weight and Haugh unit were significantly higher (*p* < 0.001) in the HFE group, followed by the MFE and lastly, the LFE group. Yolk weight, yolk width, yolk:albumen ratio and albumen width were, however, higher (*p* < 0.001) in the LFE group compared to the HFE group. There were no significant differences in the yolk colour, pH of the yolk and albumen, as well as the shell characteristics across the three FE groups.

### 3.5. Amino Acid Composition

The differences in amino acid composition from the HFE and LFE hen groups are shown in Table 3. Albumen from the HFE hen group was seen to have a higher concentration of all amino acids investigated compared to the LFE groups.

### 3.6. Nutrient Digestibility

The differences in nutrient digestibility parameters are presented in Table 4. There was no significant difference in the gross energy retention, nitrogen retention, dry matter digestibility and the apparent metabolisable energy of hens from the varying FE groups; however, dry matter intake and output were lower (*p* < 0.0001) in the HFE and MFE cohort compared to the LFE groups.

## 4. Discussion

Retrospective ranking of the laying hen cohort based on FCR in early-lay revealed that differences in FCR were primarily driven by variation in voluntary feed intake, with the hens ranked as HFE having 9.7% and 22.7% lower voluntary appetite compared with MFE and LFE hens, respectively. In contrast, the rate of lay and egg weight was a less important factor influencing FCR rankings. At mid-lay, hens (ranked based on FCR from the 25–30-week period) maintained their FCR group differences until at least 40 weeks of age. The hens from the LFE and MFE cohort consumed 11% and 4% more feed respectively, compared to the hens in the HFE group. Egg weight and rate of lay were numerically lower in the LFE group but not significantly different across groups during the 35- to 40-week period. The FCR of the LFE hens was higher (worse) than that of the HFE hens by 20.3%, and this was because the feed intake was lower and egg mass was higher in the HFE cohort. Taken together, these findings suggest that feed intake is the major factor responsible for the differences in FE between hens in this study. Further, the present study shows that once hens commence lay, feed intake, rate of lay, egg weight and egg mass are relatively stable, as the differences between groups established in early-lay remained divergent at 40 weeks. The initial and final body weights of the hens in the mid-laying stage differed across the three groups. At both 30 and 40 weeks, the birds in the LFE group were heavier than those in the MFE and HFE group, similar to the findings previously reported by Akter et al. [12]. Consequently, body weight change between 35 and 40 weeks was significantly higher in the LFE group compared to the MFE and HFE group. The relationship between feed intake and hen body weight across the three FE groups agrees with the reports from Arthur et al. [24], which concluded that variation in feed intake is associated with variation in hen body weight and together is associated with differences in maintenance requirements. From this study, the average body weight for hens between 30 to 40 weeks surpassed the breed expectation range by 84 g (1945 g in the present study vs. 1861 g according to the ISA Brown guide [17] for hens between 30 and 40 weeks); however, heavier hens did not have an increased rate of lay nor produce bigger eggs, which is usually a reason for maintaining higher body weights during laying periods by egg producers. The age of the hen at first egg lay has a significant effect on the weight of the first and subsequent eggs, where hens that begin to lay at an earlier age tend to have a lower mean egg weight [25]. In the current study, the LFE hens, which were heavier birds, came into lay six days earlier and laid smaller first eggs. In hens, ovulation is governed by body lipid/energy reserves and will not commence until a threshold is reached. The LFE hens likely reached this threshold sooner due to greater body weight and therefore produced their first egg earlier than the HFE hens.

Egg abnormalities constitute an inefficiency for the egg industry. The differences in egg abnormalities formed in the early-lay stage, between 25–30 weeks, continued into the mid-lay stage, between 35–40 weeks, with eggs from the LFE group having more abnormalities compared to HFE hens. Of the egg abnormalities recorded between 35–40 weeks of age, dirty and soft-shelled eggs accounted for the prominent abnormalities observed. The eggs from the LFE hen cohort had 7.2% and 74.5% more dirty eggs compared to the MFE and HFE group, while LFE hens had 93% more soft-shelled eggs compared to both the MFE and HFE groups. It is difficult to account for why LFE hens produced more dirty eggs; however, during the nutrient retention study, it was observed that LFE hens produced more faecal matter in line with greater feed consumption, and this may be a contributory factor. The implications are that in addition to having poor FE, the hens from the LFE group also produced fewer saleable eggs.

External egg characteristics are an important measure of quality and also affect consumer preference. Apart from the egg width, external egg measurements were unaffected by the FE groupings. The smaller egg width in the LFE hen cohort suggests narrower eggs which are not desirable as they negatively influence egg shape index, egg grading and packaging [26]. Internal egg measurements were strongly influenced by FE group. The higher albumen height observed in the HFE group in the study indicates a better albumen quality compared to the MFE group and, lastly, the LFE group. This is similar to findings from Wang et al. [27], who found that eggs with a higher albumen height had a stronger defence against bacterial invasion. When albumen height was corrected for egg weight, the Haugh unit obtained in the HFE cohort was 9.7% higher than that in both the MFE and LFE groups, suggesting that eggs from the HFE hens could potentially provide more protection against environmental shock and microbial invasion to the yolk. Further, it suggests that the albumen from the HFE hens will thin slower and thus have longer storage potential compared to the eggs from the MFE and LFE groups of hens [28]. The higher albumen index obtained in the HFE group, and the higher albumen volume as observed in the HFE hen cohort, coupled with the functions of the chalazae, which maintains the yolk in the centre of the egg, suggests a well-rounded coverage of thick albumen around the yolk, ensuring maximum protection of the egg yolk against bacterial penetration and protecting it from physical shock damage and bacterial invasion [29]. The bigger yolks observed in the LFE hen cohort in the current study may result in a reduction in the strength of the vitelline membrane, thus suggesting a higher occurrence of yolk rupture [30]. During storage, this may impact negatively on the egg, predisposing it to bacterial invasion compared to eggs from the HFE group. The higher yolk:albumen ratio observed in the LFE group when compared to the MFE and HFE groups indicates that excess lipids not used for egg production or body maintenance were stored up as body fat or deposited in the yolk [28], and this may be linked to the higher feed intake and body weight. Further, body fat and lipid synthesis require more energy from the feed compared to albumen production; thus, the differences in appetite and body weight between LFE and HFE hens may also be credited to the increased yolk weight of eggs in the LFE group compared to the HFE group. This is supported by the study of Hurnik et al. [31], who reported a positive correlation between yolk weight and feed consumption. The wider yolk width observed in the LFE group (*p* = 0.03) compared to those from the HFE groups suggests an increased water content in the LFE yolks, possibly a consequence of liquid from the albumen moving through a more permeable vitelline membrane and ultimately leading to a reduction in yolk quality [28]. During storage, eggs with a wider width and higher water infiltration will lead to faster egg deterioration. Yolk index, a measure of the spherical nature of yolk and an indicator of quality in eggs, was significantly higher in the HFE group compared to the LFE group. A higher yolk index implies an increase in quality [32], and just like most egg quality parameters, the yolk index worsens during storage. Yolk colour is enhanced through the consumption of dietary-derived pigments, such as carotenoids. There was no difference seen in the yolk colour across the three groups of hens. The lack of difference observed in the yolk colour score across the three groups in this study suggests that increased feed intake, as recorded in the LFE hen group, did not increase the yolk colour score. The lack of differences observed for all eggshell parameters across the three FE groups in the present study indicates that among individual ISA Brown hybrid hens in the mid-stages of lay, an increase in feed intake and body weight did not influence shell quality.

The concentrations of all amino acids profiled from the egg albumen in the current study were significantly higher in the HFE groups compared to the LFE groups. Essential amino acids are an indicator of protein quality [33], and egg proteins from the albumen have been reported to show antioxidant properties [34]. The amino acid profile and bioavailability in the diet is an important determinant of the amino acid profile of the egg. In this study, all hens were fed the same composition of diet; however, the LFE hens consumed more feed when compared with the HFE hens. It is therefore counter-intuitive that there would be lower concentrations of amino acids in the albumen of the LFE birds. Antioxidant compounds have been reported to play important roles in increasing the quality and the shelf life of products due to their ability to reduce oxidation [35]. The higher levels of phenylalanine and tyrosine seen in the eggs from the HFE group and supported by the finding from Nimalaratne et al. [36] suggest a higher antioxidant capacity compared to eggs from the LFE group. This study, therefore, concludes that increased efficiency of feed nutrients by the hens in the HFE group is associated with increased amino acid deposition in the albumen. Individual birds may differ in their ability to digest feed [37] due to variation in the size of the gastrointestinal tract, digesta retention time and digestive enzyme activity. Although the nutrient retention measurements across the three efficiency groups in the current study did not vary, the LFE hens with a larger feed intake rate produced more dry matter output compared to the HFE cohort. This is similar to studies that reported that despite adequate nutrient levels and energy density in offered diet, inefficient hens were not efficiently utilising the nutrients and were consuming a greater amount to compensate for the deficiencies [38,39].

## 5. Conclusions

This study has shown that feed efficiency status, once attained in early lay, is maintained until at least 40 weeks of age. Hens ranked as HFE had a lower appetite and BW, commenced onset of lay later and produced bigger first eggs compared to LFE hens, which consumed more feed, had greater bodyweight and laid smaller eggs earlier. Whereas the eggshell quality was comparable between FE groups, egg abnormality was higher among eggs from LFE hens. From this study, nutrient retention was not different between groups; however, feed efficiency and egg quality are associated with HFE hens producing eggs with higher albumen quality.

## Figures and Tables

**Table 1:** Average performance variables of individual ISA Brown hens ranked based on FCR during the preliminary ranking period (25–30 weeks) and their subsequent performance in follow up experimental period (35–40 weeks), *n* = 48 hens per FE group.

Hen Age	FE	FCR	EP	EW	EM	ADFI	IBW	FBW	BWC
Preliminary ranking period (25–30 weeks)	HFE	1.83	98.8	63.5	62.0	113	1760	1923	164
	MFE	2.05	98.8	61.9	60.3	124	1801	2016	215
	LFE	2.39	97.9	60.8	58.3	138	1877	2159	282
Experimental period (35–40 weeks)	HFE	1.77 ^a^	98.0	64.8	63.6 ^a^	113 ^a^	1923 ^a^	1966 ^a^	43.1 ^a^
	MFE	1.89 ^b^	98.0	63.5	62.2 ^b^	118 ^b^	2016 ^b^	2055 ^b^	38.3 ^a^
	LFE	2.13 ^c^	96.4	62.9	60.6 ^b^	128 ^c^	2154 ^c^	2253 ^c^	98.5 ^b^
	SEM	0.10	0.60	0.58	0.69	0.35	24	29	13
	*p*-value	<0.0001	0.089	0.06	0.01	<0.0001	<0.0001	<0.0001	0.001

FE: Feed efficiency; HFE: High feed efficiency; MFE: Medium feed efficiency; LFE: Low feed efficiency. SEM: Standard Error Mean. ^abc^: Means within columns with a different superscript are significantly different at the 5% level of significance. EP: Egg produced per hen per day (%); EW: Egg weight (g); EM: Egg mass (g); ADFI: Average daily feed intake (g); FCR: Feed to egg conversion ratio = ADFI/Egg mass; IBW: Initial Body Weight (g); FBW: Final Body Weight (g); BWC: Body weight change (g); Egg mass = Egg weight × egg production/100 (g).

**Table 2:** Internal and external egg quality characteristics of eggs from individual ISA Brown hens within 35–40 weeks of age, based on differences in feed efficiency.

	Hen Feed Efficiency Group (*n* = 15 per FE Group)				
Egg Quality Traits	HFE	MFE	LFE	SEM	*p*-Value
Egg weight (g)	64.5	62.6	61.7	0.811	0.055
Egg width (mm)	44.7 ^a^	43.9 ^b^	43.9 ^b^	0.19	0.011
Egg height (mm)	57.2	57.3	56.5	0.36	0.284
Shape index (%)	78.1	76.8	77.7	0.44	0.112
Albumen height (mm)	11.1 ^a^	10.2 ^b^	8.8 ^c^	0.23	<0.0001
Albumen width (mm)	66.1 ^b^	65.5 ^b^	68.1 ^a^	0.85	<0.0001
Albumen index (%)	16.6 ^a^	15.7 ^a^	13.0 ^b^	0.50	<0.0001
Albumen weight (g)	38.8 ^a^	38.1 ^b^	37.4 ^c^	0.20	<0.0001
Albumen weight (%)	61.8 ^a^	60.5 ^b^	59.3 ^c^	0.31	<0.0001
Haugh unit	103 ^a^	99 ^b^	93 ^c^	0.98	<0.0001
Albumen pH	8.0	7.9	8.1	0.03	0.193
Yolk height (mm)	17.7	17.5	17.4	0.10	0.179
Yolk width (mm)	37.6 ^b^	38.1 ^b^	39.0 ^a^	0.17	<0.0001
Yolk index (%)	46.7 ^a^	46.1 ^a^	45.1 ^b^	0.32	0.004
Yolk weight (g)	15.0 ^b^	15.4 ^b^	16.3 ^a^	0.17	<0.0001
Yolk weight (%)	23.9 ^b^	24.5 ^b^	25.9 ^a^	0.27	<0.0001
Yolk pH	5.9	5.9	6.0	0.03	0.531
Yolk:albumen ratio	0.38 ^c^	0.40 ^b^	0.43 ^a^	0.006	<0.0001
Yolk colour (DSM)	11.5	11.3	11.4	0.094	0.416
Shell weight (g)	6.42	6.41	6.26	0.07	0.251
Shell thickness (mm)	0.41	0.41	0.41	0.002	0.493
Shell weight (%)	10.0	10.3	10.2	0.13	0.292
Shell breaking strength (g)	4607	4818	4995	146.91	0.186

HFE: High feed efficiency; MFE: Medium feed efficiency; LFE: Low feed efficiency; SEM: Standard Error of Mean; ^abc^: Means within columns with different superscript are significantly different at the 5% level of significance. Egg shape index = egg width/egg length × 100; Albumen percentage = albumen weight/egg weight × 100; Albumen index = Albumen height/albumen width × 100; Yolk percentage = Yolk weight/egg weight × 100; Yolk index = Yolk height/yolk width × 100; Yolk: Albumen ratio = Yolk weight/Albumen weight; Shell percentage = Shell weight/egg weight × 100.

**Table 3:** The concentration of amino acids in egg albumen collected from high and low feed efficient ISA Brown hens in the mid stages of lay.

	FE Group (*n* = 10 per FE group)			
Amino Acid, mg/g Freeze-Dried Albumen	HFE	LFE	SEM	*p*-Value
Histidine	21.6	20.3	0.16	<0.0001
Serine	60.9	57.9	0.47	0.0003
Arginine	51.2	48.4	0.42	0.0002
Glycine	31.6	29.7	0.27	<0.0001
Aspartic acid	119.4	114.1	0.90	0.0014
Glutamic acid	91.3	86.7	0.85	0.0006
Threonine	40.6	38.1	0.38	0.0002
Alanine	51.7	49.1	0.41	0.0003
Proline	32.3	30.6	0.24	0.0001
Lysine	61.4	57.6	0.70	0.0012
Tryosine	33.9	31.9	0.34	0.0006
Methionine	35.9	34.3	0.25	0.0003
Valine	62.3	59.2	0.48	0.0003
Isoleucine	48.8	46.6	0.35	0.0003
Leucine	77.5	73.8	0.54	0.0001
Phenylalanine	55.2	52.4	0.39	<0.0001

HFE: High feed efficiency; LFE: Low feed efficiency; SEM: Standard Error of Mean.

**Table 4:** Apparent metabolisable energy (AME), nitrogen retention and digestibility rate of individual ISA Brown hens in the mid stages of lay, based on differences in their FE. (*n* = 20 per FE group).

Measurements	HFE	MFE	LFE	SEM	*p*-Value
Dry matter intake (g/day)	102.1 ^a^	105.5 ^a^	117.2 ^b^	2.08	<0.0001
Dry matter output (g/day)	23.5 ^a^	24.8 ^a^	28.6 ^b^	0.79	<0.0001
Dry matter retention (%)	76.8	76.5	75.5	0.60	0.3287
Nitrogen retention (%)	57.8	57.9	56.3	1.25	0.6020
Gross energy retention (%)	79.5	79.0	79.0	0.49	0.4902
Apparent metabolisable energy (MJ/kg) of DM	12.1	12.0	12.05	0.07	0.4798
Apparent metabolisable energy (MJ/kg) corrected for N	11.9	12.0	11.8	0.06	0.3597

HFE: High feed efficiency; MFE: Medium feed efficiency; LFE: Low feed efficiency; SEM: Standard Error of Mean; N: Nitrogen; DM: Dry matter. ^ab^ Means within columns with different superscript are significantly different at the 5% level of significance.

## Data Availability

The data presented in this study are available on request from the corresponding author.
